# Management of Extra-articular Distal Femoral Fractures Through Closed Reduction and Internal Fixation Using Minimally Invasive Plate Osteosynthesis Versus Retrograde Intramedullary Nailing: A Comparative Study

**DOI:** 10.7759/cureus.99650

**Published:** 2025-12-19

**Authors:** Mohammad Monir, Alaa Mohey Eldin Solyman, Khaled Mohammed Abdel Halim, Hisham Misbah, Hassan Hussain, Khaled Altaraman, Mohammed Hassan Suliman, Ahmed Sayedin, Mahmoud Zeid, Ahmed Al-feeshawy

**Affiliations:** 1 Orthopedics and Trauma, General Organization for Teaching Hospitals and Institutes, Cairo, EGY; 2 Orthopedics and Trauma, Cairo University Hospitals, Cairo, EGY; 3 Orthopedics and Trauma, Banha University Hospitals, Banha, EGY; 4 Orthopedic Surgery, King Fahad Military Medical Complex, Dammam, SAU; 5 Orthopedics and Trauma, King Fahad Military Medical Complex, Dammam, SAU; 6 Radiology, Klinikum Ingolstadt, Ingolstadt, DEU; 7 Trauma Surgery, Krankenhaus St. Josef, Schweinfurt, DEU

**Keywords:** comparative study, distal femur fracture, functional outcome, minimally invasive plate osteosynthesis (mipo), retrograde intramedullary nailing

## Abstract

Background: Distal femoral fractures (DFFs) account for a small proportion of femoral fractures and can be challenging to manage. The goal of treatment is to restore alignment, length, and function while minimizing soft-tissue disruption. Two common fixation options are minimally invasive plate osteosynthesis (MIPO) and retrograde intramedullary nailing (RIMN). This study prospectively compared the outcomes of both techniques in extra-articular DFFs.

Materials and methods: A prospective comparative study was conducted on 40 adult patients with extra-articular DFFs (Arbeitsgemeinschaft für Osteosynthesefragen/Orthopaedic Trauma Association 33-A (AO/OTA 33-A)). Patients were allocated to two treatment groups: MIPO (n = 20) and RIMN (n = 20) according to the surgeon’s preference. Outcomes included time to radiological union and range of motion. Functional results were assessed using the Knee Society Score and Lysholm Knee Score at three and six months, and complications were recorded. Statistical analyses were performed using independent t-tests and Fisher’s exact test, with significance set at p < 0.05.

Results: Both groups showed significant functional improvement over time. At six months, good-to-excellent results were seen in 80% of MIPO and 85% of RIMN patients. RIMN patients achieved earlier full weight-bearing (average 6.2 weeks vs. 8.3 weeks; mean difference −2.1 weeks (95% CI −2.9 to −1.3; t(38) = −4.65, p < 0.001). and slightly better knee flexion at final follow-up (112° ± 7.5 vs 107° ± 8.2); mean difference 5.0° (95% CI 1.2 to 8.8; t(38) = 2.68, p = 0.011). However, anterior knee pain was more frequently reported after RIMN (20% vs. 5%), although this difference was not statistically significant (p = 0.342). Two superficial infections occurred in the MIPO group (10%). The mean time to union was 14.3 ± 1.9 weeks (MIPO) and 15.1 ± 2.1 weeks (RIMN); the mean difference was −0.8 weeks (95% CI −2.0 to 0.4; t(38) = −1.26, p = 0.214), and no reoperations were required. This prospective study adds focused evidence on extra-articular (AO/OTA 33-A) fractures, which remain underrepresented in comparative research.

Conclusions: Both MIPO and RIMN are reliable methods for extra-articular DFFs, providing comparable rates of union and excellent functional outcomes. While a broader range of final coronal alignment was observed in the RIMN group, this did not compromise stable union or functional recovery. RIMN offers faster rehabilitation and earlier weight-bearing, but with a higher reported incidence of anterior knee pain. MIPO is associated with lower knee morbidity and may be a suitable alternative, particularly for patients in whom minimizing anterior knee symptoms is a key concern, despite its slower rehabilitation pathway. The choice should be tailored to the fracture pattern, bone quality, and patient needs.

## Introduction

Modern high-velocity transportation and contemporary lifestyles have rendered the distal femur particularly susceptible to trauma. Distal femoral fractures (DFFs) often constitute complex injuries that pose significant management challenges and may lead to considerable long-term disability. Despite advancements in surgical techniques and the evolution of internal fixation systems, the treatment of DFFs remains demanding across various clinical contexts [1].

The prevalence of DFFs increases with age, comprising nearly 1% of all skeletal injuries and representing 4% to 6% of femoral fractures. However, among elderly patients, they are the second most prevalent type of femoral fracture. Young cases often experience these fractures due to high-velocity trauma. These fractures are compounded by osteoporosis, significant comminution, and intra-articular extension [2].

Advancements in surgical techniques have introduced various fixation methods, including buttress plates, dynamic condylar screws (DCS), locking plates, and intramedullary nails (IMN), to facilitate early mobilization [2,3]. Modern fixation strategies emphasize biological principles, preservation of the periosteal blood supply, and minimally invasive approaches, such as less-invasive stabilization system (LISS) plating, minimally invasive plate osteosynthesis (MIPO), and retrograde intramedullary nailing (RIMN) [3].

Biomechanical research, primarily using cadaver studies, has focused on achieving optimal stability for these fractures and on evaluating the efficacy of locking metal plates compared with RIMN. While securing lateral column support is generally straightforward, given that most fixation devices are applied to the lateral femoral cortex, achieving adequate reduction of medial comminution remains challenging with lateral-only constructs [3].

Metaphyseal comminution poses difficulties for traditional plate fixation. The locked compression plate (LCP) creates a fixed-angle construct and allows for plate installation without bone contact, preserving the underlying periosteal blood supply. It thus can be utilized in metaphyseal comminution, and the pull-out strength of locking screws greatly exceeds that of standard screws, making it challenging for a single screw to disengage or fail unless the whole construct fails as a single unit. This enhances fixation reliability, especially in osteoporotic bone. Functioning as an internal fixator, the LCP is well-suited for use in MIPO techniques [4,5].

Küntscher introduced the notion of IMNs, which has since been significantly refined. IMN has emerged as a novel therapeutic approach for treating DFFs. They provide superior "biological" support compared to plates, function as load-sharing appliances, facilitate enhanced soft-tissue preservation, demonstrate lower infection rates, and offer effective fixation in osteoporotic bone. Clinical outcomes have demonstrated union rates approaching 99%, with patients typically achieving a postoperative knee range of motion (ROM) near 130° [1].

Although the burden-sharing device for intramedullary applications greatly facilitates premature packing and offers a compelling remedy for these fractures, systemic problems, persistent knee discomfort, and possible knee arthritis remain challenges for nailing procedures [6].

When comparing LISS plating and RIMN, it is observed that weight-bearing following LISS plating takes longer to prevent plate breakage. In contrast, prompt loading is feasible after the nailing procedure. To date, there are no statistically meaningful differences between the nailing and LISS communities regarding osseous healing, union rates, and post-surgical complications [6].

Given these considerations, the optimal fixation method for extra-articular DFFs remains debated. This study was designed to prospectively compare the short-term outcomes of MIPO with distal femoral locking plate (DFLP) and RIMN.

Although several clinical studies have compared plating and nailing techniques, many combined both intra-articular and extra-articular fractures or used mixed fracture classifications. There is limited evidence focusing exclusively on extra-articular (Arbeitsgemeinschaft für Osteosynthesefragen/Orthopaedic Trauma Association 33-A (AO/OTA 33-A)) fractures. Therefore, this study was designed to provide prospective comparative data on functional and radiological outcomes using MIPO and RIMN for these fractures.

This prospective comparative study aimed to evaluate the radiological and functional outcomes of extra-articular DFFs treated with MIPO versus RIMN.

## Materials and methods

Study design and setting

This was a prospective, non-randomized comparative cohort study conducted at Kasr Al-Ainy University Hospitals and Ahmed Maher Teaching Hospital between April and September 2023. Ethical approval was obtained from the Cairo University Faculty of Medicine Research Ethics Committee (approval number: MD-196-2023), and written informed consent was obtained from all participants. Consecutive adult patients who met the inclusion criteria were enrolled using a standardized data-collection protocol.

Participants

This prospective study enrolled 40 consecutive skeletally mature patients with closed or Gustilo-Anderson type I open extra-articular DFFs (AO/OTA types 33-A1, A2, or A3). The study included patients aged ≥18 years with isolated, extra-articular DFFs considered suitable for internal fixation using either MIPO or RIMN. Patients were excluded if they had fractures with intra-articular extension (AO/OTA types 33-B or 33-C), Gustilo-Anderson type II or III open fractures, pathological or periprosthetic fractures, or were unable to provide informed consent or comply with the minimum 6-month follow-up protocol.

Study Groups and Allocation

The total sample size of 40 (20 per group) was chosen pragmatically and was not based on a formal a priori power calculation. This was a non-randomized study. Patients were allocated to one of two treatment groups based on the operating surgeon’s clinical judgment. Group 1 (MIPO, n = 20) comprised patients treated with a DFLP using a minimally invasive approach. Group 2 (RIMN, n = 20) comprised patients treated with RIMN. All surgical procedures were performed by five senior orthopedic trauma surgeons, each proficient in both MIPO and RIMN techniques and with over 10 years of experience in orthopedic trauma. Each surgeon operated on patients in both the MIPO and RIMN groups. Allocation to MIPO or RIMN was based on the operating surgeon's preference and individualized clinical judgment, reflecting real-world decision-making. Complex cases and treatment plans were reviewed in preoperative orthopedic trauma meetings under the supervision of four senior professors. No patient was lost to follow-up, and all enrolled patients were included in the final analysis.

Rationale for Allocation

Patients were allocated according to the surgeon’s intraoperative assessment and individualized clinical judgment rather than by randomization. This pragmatic approach reflected real-world clinical practice during the study period; however, it can introduce selection bias (for example, surgeons may have preferred RIMN for specific fracture morphologies or patient profiles). To address this, we prospectively enrolled consecutive patients and compared baseline characteristics between groups; residual confounding remains possible and is acknowledged.

Operative difficulties

Both techniques were performed under fluoroscopic guidance on a radiolucent table under spinal or general anesthesia. In the MIPO technique, the main intraoperative challenges included maintaining indirect reduction through small incisions, ensuring accurate plate positioning on the distal fragment, and achieving satisfactory screw purchase in osteoporotic bone.

Insertion or exchange of locking screws through stab incisions can be challenging. Occasionally, the screw head may disengage from the screwdriver, requiring longer operative time, increased fluoroscopic exposure, and, sometimes, wound enlargement. In such situations, we found the following helpful maneuver: A 2-0 Vicryl suture is tied to the screw head before seating; once the screw is appropriately seated, the suture is cut deeply and removed. This simple measure helps retrieve and control screws through limited incisions while minimizing additional soft-tissue trauma.

In the RIMN technique, several technical issues were encountered and managed. During reduction, applying straight-line traction to the foot may worsen distal-segment flexion due to the gastrocnemius pull. We therefore provided traction with the limb supported at approximately a 30° angle over a triangular support to reduce this flexion deformity.

To preserve nail-bone alignment during distal interlocking, keep the drill bit engaged in the hole until the locking screw is ready for insertion. Additionally, using a locking screwdriver or securing a silk suture around the screw head helps prevent the screw from being displaced into surrounding soft tissues.

Remember that the distal femur narrows from posterior to anterior; on a standard anteroposterior fluoroscopic view, locking screws can appear to lie within bone but actually protrude anteriorly. If a screw appears to project beyond the cortex on anteroposterior imaging, it is likely too long and could cause pain or contribute to heterotopic ossification. To assess true screw length, obtain anteroposterior views at differing limb rotations (e.g., with the lower extremity internally rotated ~30° and externally rotated ~20°) before final screw trimming.

Postoperative management

All patients received intravenous antibiotics for 48 hours, followed by a one-week oral course and subcutaneous enoxaparin (40 mg daily) until full mobilization. For physiotherapy, quadriceps and hamstring isometric exercises and passive knee motion were initiated as tolerated within the first postoperative week. Active mobilization progressed gradually to achieve a full, pain-free ROM.

For weight-bearing, the progression to full weight-bearing was guided by radiographic evidence of callus formation for all patients. However, the rehabilitation protocol was tailored to each implant's inherent stability. Patients in the RIMN group were encouraged to bear weight as tolerated with crutch support from the early postoperative period, progressing to full weight-bearing as soon as clinically and radiographically appropriate (typically by 6-8 weeks). In contrast, patients in the MIPO group followed a more cautious protocol, initiating partial weight-bearing at approximately 6-8 weeks and advancing to full weight-bearing only after a clear bridging callus was evident, usually by 10-12 weeks. These distinct rehabilitation protocols were designed according to the inherent biomechanical properties of each fixation construct.

Outcome measures

Primary outcomes were time to radiological union and final knee ROM. Radiological union was defined as bridging callus across ≥3 cortices on two orthogonal radiographs. ROM was measured using a standard goniometer by an independent assessor. Secondary outcomes included operative time, the Knee Society Score (KSS) and Lysholm Knee Score (LKS), and complications (infection, delayed union, implant failure, and anterior knee pain).

Statistical analysis

Statistical analyses were performed using SPSS Statistics version 28 (IBM Corp. Released 2021. IBM SPSS Statistics for Windows, Version 28.0. Armonk, NY: IBM Corp.). Continuous variables were inspected for normality using the Shapiro-Wilk test and for homogeneity of variances using Levene’s test; when Levene’s p < 0.05, the Welch t-test was used. Normally distributed continuous variables are expressed as mean ± standard deviation (SD) and compared with independent-samples t-tests (or Welch t-tests when appropriate). Non-normal data are presented as median (IQR) and compared using the Mann-Whitney U test. Ordinal outcomes (KSS and LKS categories) were compared within groups over time using the Marginal Homogeneity test and between groups using the Mann-Whitney U or Chi-square test. Categorical variables with expected cell counts <5 were analyzed with Fisher’s exact test. Effect sizes are reported as mean differences with 95% confidence intervals (CIs) for continuous variables and relative risks (RRs, 95% CIs) for categorical outcomes; corresponding test statistics (t, χ², or z) are stated. A two-sided p < 0.05 was considered statistically significant.

## Results

Demographics

Forty consecutive patients were enrolled: 20 treated with MIPO and 20 with RIMN. The two groups were comparable in baseline age, sex, and AO/OTA fracture type 33-A1-A3 (p > 0.05 for all). No patients were lost to follow-up. Demographic characteristics were comparable between groups. Table 1 shows that the cohort had a mean age of 52.9 ± 12.8 years, with male predominance (55%). Road traffic accidents accounted for 60% of injuries, and falls for 40%; also, fracture classification included A1 (42.5%), A2 (22.5%), and A3 (35%) patterns. Baseline variables, including age, sex, mechanism of injury (road-traffic accident vs. fall), affected side, and comorbidities, were compared using independent t-tests or Chi-square/Fisher’s exact tests as appropriate; no statistically significant differences were identified (all p > 0.05)

**Table 1:** Baseline characteristics of patients in both groups (MIPO vs. RIMN) Data are presented as mean ± SD or n (%). P-values are from an independent-sample t-test for continuous variables and a chi-square test for categorical variables, unless expected cell counts <5, in which case Fisher’s exact test was used. AO/OTA: Arbeitsgemeinschaft für Osteosynthesefragen/Orthopaedic Trauma Association classification, MIPO: minimally invasive plate osteosynthesis, RIMN: retrograde intramedullary nailing, SD: standard deviation

Characteristic	MIPO (n = 20)	RIMN (n = 20)	p-value
Age (years), mean ± SD	54.7 ± 11.7	51.0 ± 13.8	0.34
Male, n (%)	12 (60%)	9 (45%)	0.34
AO/OTA classification, n (%)			0.92
A1	8 (40%)	9 (45%)	
A2	5 (25%)	4 (20%)	
A3	7 (35%)	7 (35%)	

Outcomes

Operative Parameters

The mean operative duration was 83 ± 25 minutes in the MIPO group and 92 ± 39 minutes in the RIMN group. The mean difference was −9 minutes (95% CI −29.3 to 11.3; t(34) = −0.87, p = 0.39), suggesting comparable intraoperative times between both techniques. Although RIMN involved slightly longer fluoroscopy use, the overall surgical efficiency was similar across groups.

Radiological Union and Radiographic Alignment

The mean time to union was 14.3 ± 1.9 weeks in the MIPO group and 15.1 ± 2.1 weeks in the RIMN group. The mean difference was −0.8 weeks (95% CI −2.0 to 0.4; t(38) = −1.26, p = 0.214), indicating no statistically significant difference between the two fixation methods. Radiographic bridging of three or more cortices was observed in all patients within six months, with delayed union occurring in one MIPO case and two RIMN cases.

Quality of reduction and maintenance: The quality of the initial postoperative reduction and its maintenance were assessed radiographically. Coronal alignment was measured using the anatomical lateral distal femoral angle (mLDFA) on anteroposterior views, and sagittal alignment was assessed via the posterior cortical angle on lateral views.

Immediate postoperative alignment: The mean mLDFA was 85.2° ± 3.1° (range: 81° to 90°) in the MIPO group and 84.8° ± 3.9° (range: 78° to 91°) in the RIMN group (p = 0.71).Two patients in the RIMN group (cases 4 and 6) presented with postoperative coronal alignments of 2° and 12° of valgus, respectively, which fell outside the typical physiological range of 5-10°. Both alignments remained stable throughout follow-up and did not prevent an uneventful union. All other patients in both groups exhibited coronal alignment within the normal valgus range. Sagittal alignment was comparable between groups (82.5° ± 4.5° MIPO vs 83.1° ± 4.2° RIMN, p = 0.65).

Maintenance of reduction: Alignment was well-maintained in both groups. The mean change in mLDFA from immediate postoperative to final union radiographs was negligible (0.4° ± 0.8° MIPO vs 0.5° ± 1.1° RIMN, p = 0.72). The single case with reduced valgus maintained this alignment without progression until union.

Functional outcomes

Knee Society Score

At three months, 11 of 20 (55%) patients in the MIPO group and 14 of 20 (70%) in the RIMN group achieved good-to-excellent results (RR 0.79; 95% CI 0.48-1.30; χ²(1) = 0.96; p = 0.327). At six months, rates increased to 80% (16/20) and 85% (17/20), respectively (RR 0.94; 95% CI 0.70-1.26; Fisher’s exact p = 1.00). The mean improvement from 3 to 6 months was comparable between groups (mean difference 1.5 points; 95% CI −5.6 to 8.6; p = 0.67), indicating similar functional recovery.

Within-group improvement between three- and six-month follow-ups was statistically significant in both groups, confirming postoperative functional recovery (Marginal Homogeneity test: KSS-MIPO χ² = 12.4, p < 0.001; RIMN χ² = 10.7, p = 0.001; LKS-MIPO χ² = 9.9, p = 0.002; RIMN χ² = 8.8, p = 0.003).

Lysholm Knee Score

At three months, excellent-to-good outcomes occurred in 55% (11/20) of MIPO and 65% (13/20) of RIMN patients (RR 0.85; 95% CI 0.51-1.43; χ²(1) = 0.42; p = 0.519). At six months, both groups reached 80% (16/20) excellent-to-good results (RR 1.00; 95% CI 0.72-1.38; Fisher’s exact p = 1.00). Overall improvement in LKS between three and six months did not differ significantly (mean difference 0.8 points; 95% CI −6.4 to 8.0; p = 0.81). Functional outcomes are detailed in Tables 2-3.

**Table 2:** Distribution of KSS categories at three and six months Data are n (%) per category. P-values were calculated with the chi-square test; where expected counts <5, Fisher’s exact test was used. KSS categories: excellent/good versus fair/poor. MIPO: minimally invasive plate osteosynthesis, RIMN: retrograde intramedullary nailing, KSS: Knee Society Score

		MIPO (n = 20)		RIMN (n = 20)		p-value
		Count	%	Count	%	
KSS after 3 months	Excellent and good	11	55.0%	14	70.0%	0.327
	Fair and poor	9	45.0%	6	30.0%	
KSS after 6 months	Excellent and good	16	80.0%	17	85.0%	1
	Fair and poor	4	20.0%	3	15.0%	

**Table 3:** Distribution of LKS categories at three and six months Data are n (%) per category. P-values were calculated with the chi-square test; where expected counts <5, Fisher’s exact test was used. LKS categories: excellent/good versus fair/poor. MIPO: minimally invasive plate osteosynthesis, RIMN: retrograde intramedullary nailing, LKS: Lysholm Knee Score

		MIPO (n = 20)		RIMN (n = 20)		p-value
		Count	%	Count	%	
LKS after 3 months	Excellent and good	11	55.0%	13	65.0%	0.519
	Fair and poor	9	45.0%	7	35.0%	
LKS after 6 months	Excellent and good	16	80.0%	16	80.0%	1
	Fair and poor	4	20.0%	4	20.0%	

Range of Motion

The mean knee flexion at final follow-up was 107 ± 8.2° in the MIPO group and 112 ± 7.5° in the RIMN group. The mean difference was 5.0° (95% CI 1.2 to 8.8; t(38) = 2.68, p = 0.011), indicating significantly greater flexion in the RIMN group. These findings correspond with prior studies reporting earlier return of motion after nailing. No significant differences were observed in extension lag or pain-free ROM.

Time to Full Weight Bearing

The mean time to achieve full weight-bearing was 8.3 ± 1.2 weeks in the MIPO group and 6.2 ± 1.0 weeks in the RIMN group. The mean difference was −2.1 weeks (95% CI −2.9 to −1.3; t(38) = −4.65, p < 0.001), indicating that patients treated with RIMN were able to bear full weight significantly earlier than those treated with MIPO. This earlier mobilization reflects the greater axial stability provided by RIMN and aligns with previously reported biomechanical advantages.

Complications

Postoperative complications are summarized in Table 4. Superficial infection occurred in 2/20 (10%) MIPO and 0/20 (0%) RIMN patients (Fisher’s exact p = 0.487; RR not estimable due to zero events in RIMN). Delayed union occurred in 1/20 (5%) MIPO and 2/20 (10%) RIMN (RR 2.00; 95% CI 0.20-20.34; p = 0.600). Anterior knee pain occurred in 1/20 (5%) MIPO and 4/20 (20%) RIMN (RR 4.00; 95% CI 0.49-32.7; p = 0.342). No deep infections, implant failures, or non-unions were observed.

**Table 4:** Postoperative complication rates in the MIPO and RIMN groups Data are n (%). P-values correspond to Fisher’s exact test or the chi-square test as appropriate. delayed union: lack of radiographic union by 6 months, anterior knee pain: patient-reported pain localized to the anterior knee at follow-up, superficial infection: wound infection managed non-operatively, MIPO: minimally invasive plate osteosynthesis, RIMN: retrograde intramedullary nailing

		Group I		Group II		p-value
		Count	%	Count	%	
Delayed union	Yes	1	5.0%	2	10.0%	0.6
	None	19	95.0%	18	90.0%	
Knee pain	Yes	1	5.0%	4	20.0%	0.342
	None	19	95.0%	16	80.0%	
Superficial infection	Yes	2	10.0%	0	0.0%	0.487
	None	18	90.0%	20	100.0%	

Case presentation

Case 1

**Figure 1:**
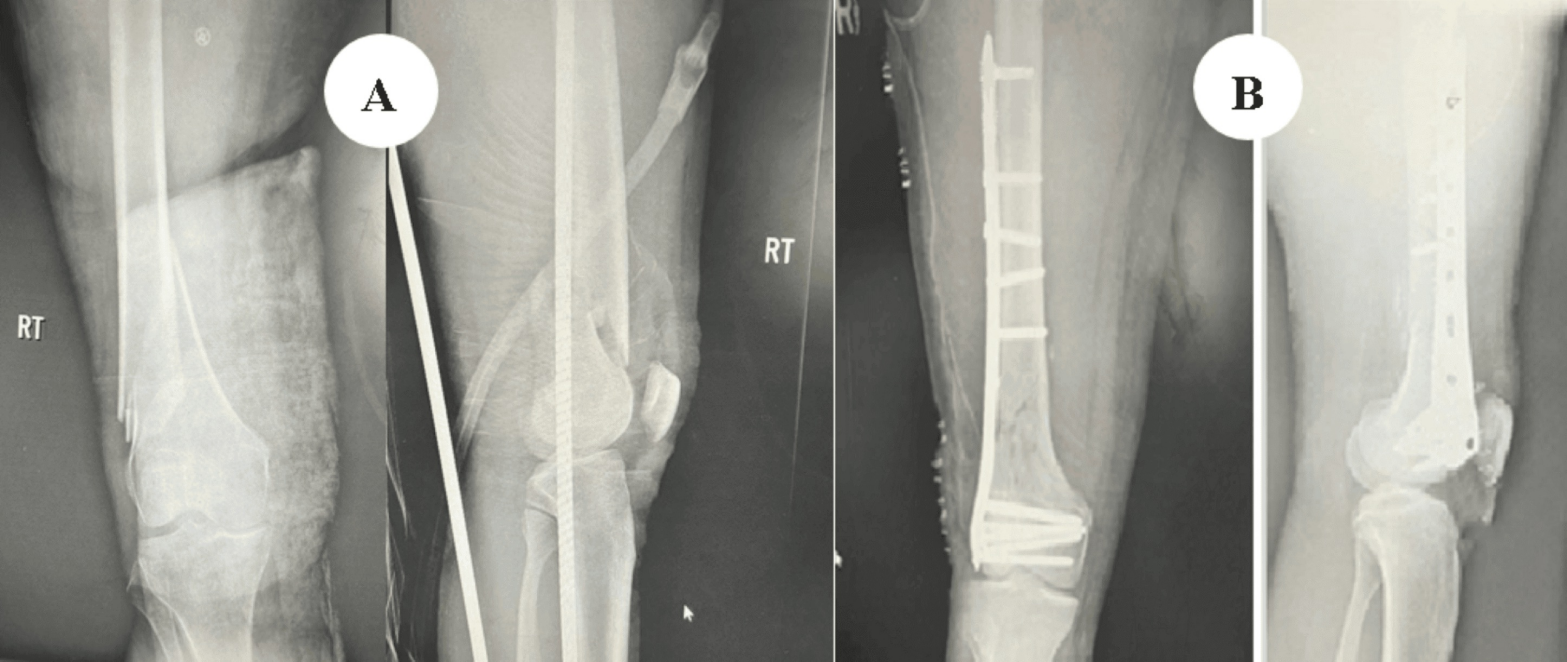
Case 1 (A) preoperative anteroposterior and lateral X-ray views and (B) immediate postoperative X-ray views

The patient was a 40-year-old male who sustained trauma following a motorbike accident. Preoperative evaluation (Figure 1A) revealed a right-sided DFF classified as AO/OTA type A1. There were no associated comorbidities. On examination, a 1 × 4 cm cut wound was noted over the anterolateral aspect of the ipsilateral knee. The time interval between injury and surgical intervention was four days.

Operative management involved indirect reduction of the fracture under C-arm guidance using both anteroposterior and lateral views, followed by fixation with a DFLP.

**Figure 2:**
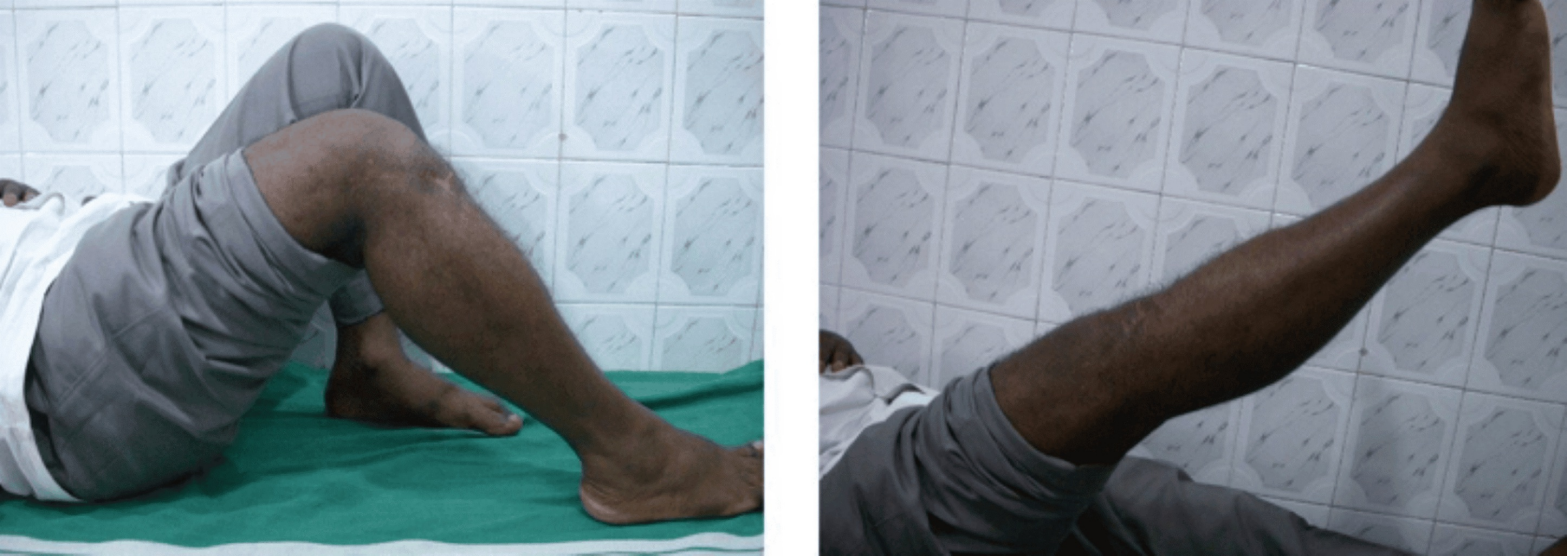
Case 1 ROM from 0° extension to 85° flexion at the six-month follow-up ROM: range of motion

**Figure 3:**
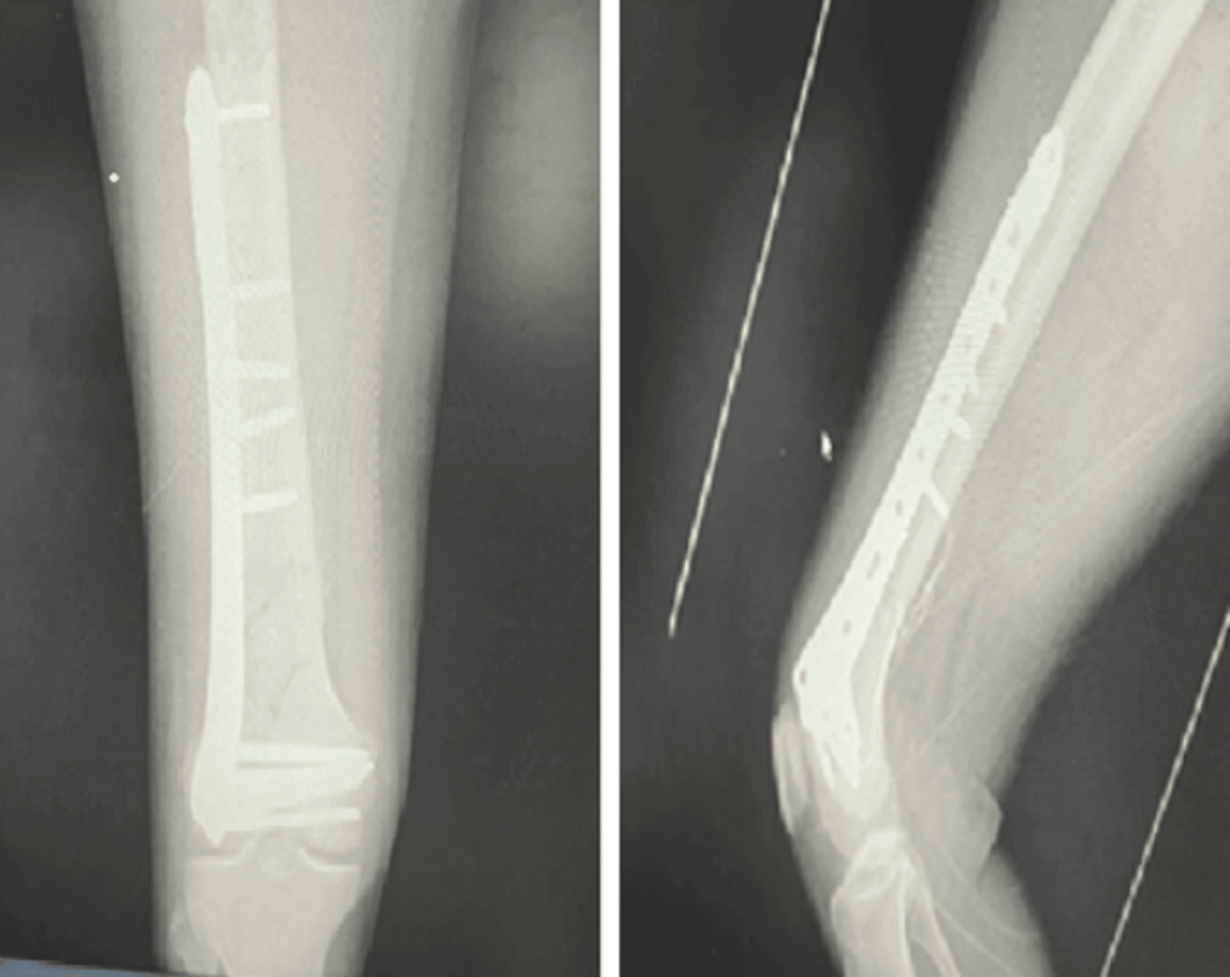
Case 1 X-ray at the six-month follow-up

Postoperatively (Figure 1B), daily wound dressings were performed. Passive and active ROM exercises were initiated on the second postoperative day. Knee joint alignment was satisfactory, with approximately 5° of valgus and no rotational deformity. Physiotherapy was commenced at three weeks postoperatively, and partial weight-bearing was initiated at six weeks. A superficial wound infection was noted on the eighth postoperative day; wound swab and culture grew *Staphylococcus aureus*, which was sensitive to co-amoxiclav. The wound became clean and dry, with no discharge, after a seven-day course of antibiotics. Radiological union was achieved at approximately ten weeks postoperatively. At follow-up, the patient had a ROM from 0° of full extension to 85° of flexion (Figure 2), was able to walk unrestrictedly, and had returned to normal activities by around 20 weeks. The total follow-up period was approximately six months (Figure 3).

Case 2

**Figure 4:**
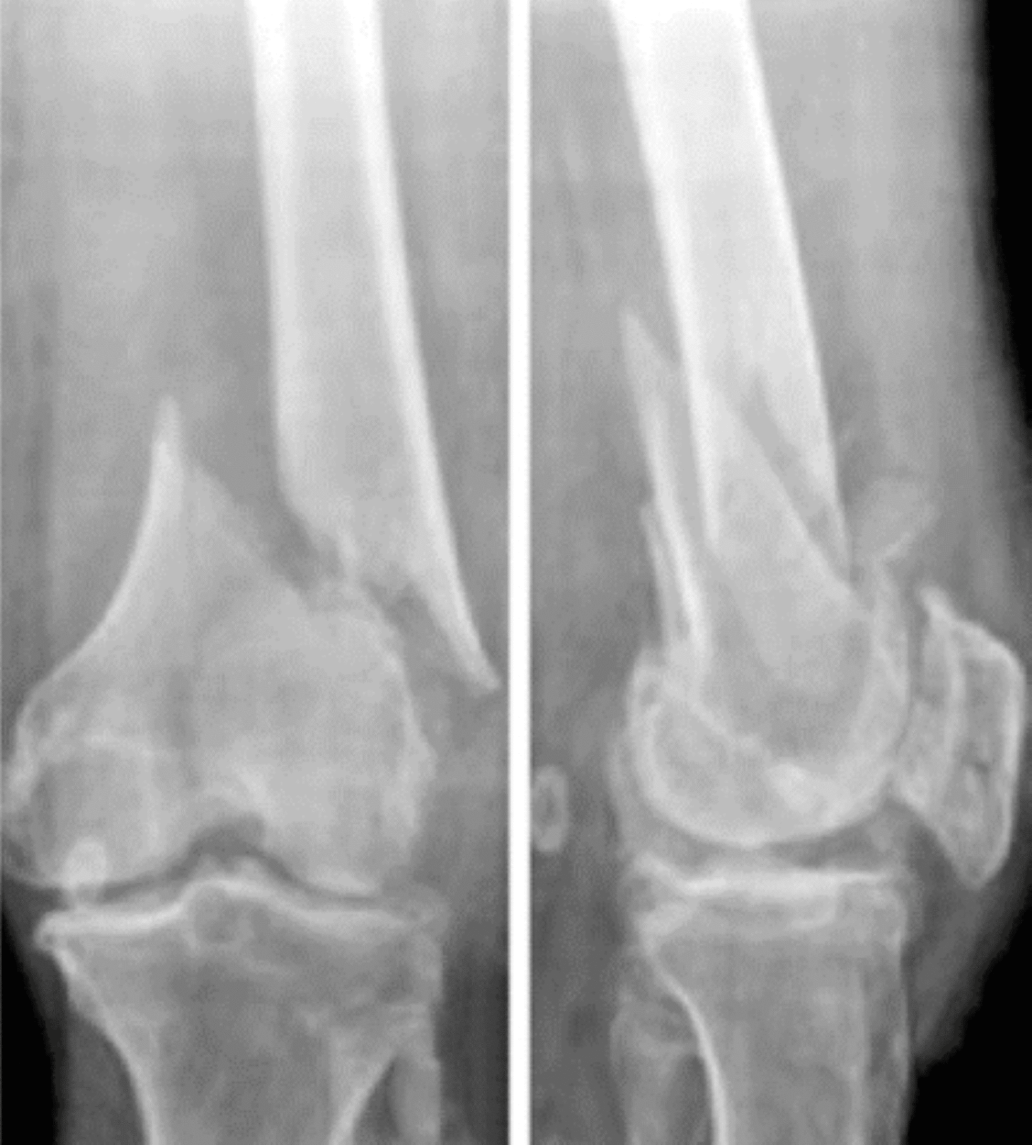
Case 2 preoperative X-rays: anteroposterior and lateral views

The patient was a 65-year-old male who sustained trauma following a motorcar accident. Preoperative evaluation (Figure 4) revealed a left-sided DFF classified as AO/OTA type A2. The patient had a history of diabetes mellitus, and no other injuries were associated with the trauma. The time interval between injury and surgery was three days.

Operative management involved indirect reduction of the fracture under C-arm guidance in both anteroposterior and lateral views, followed by fixation with a DFLP.

**Figure 5:**
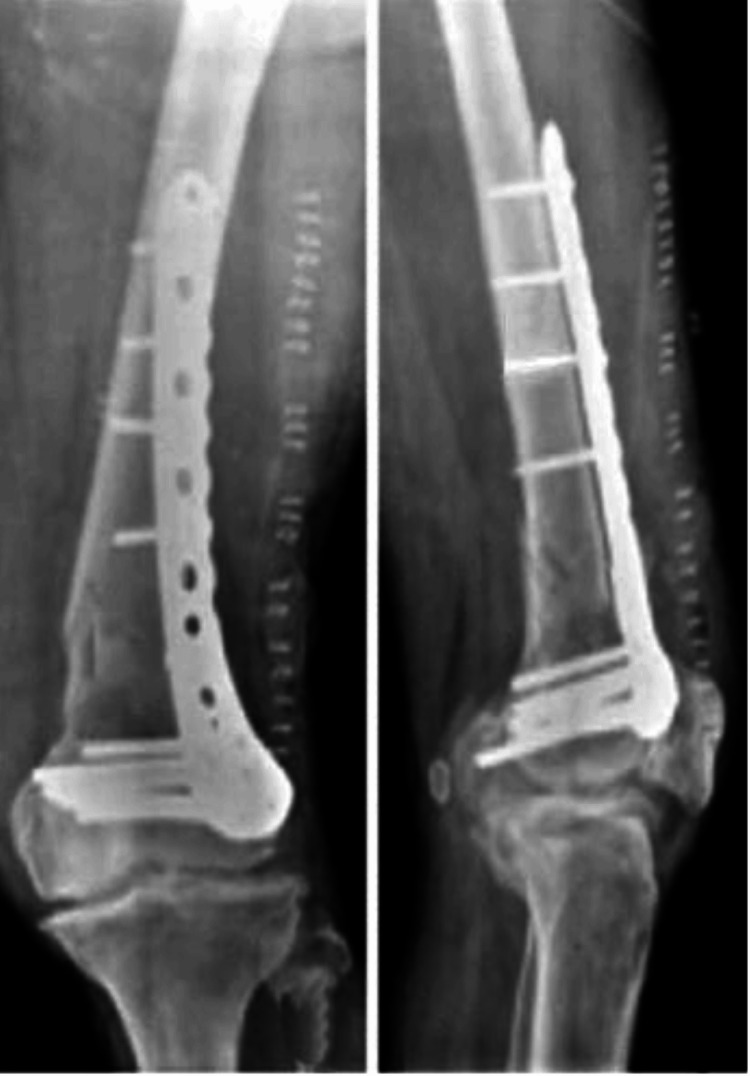
Case 2 immediate postoperative X-ray

**Figure 6:**
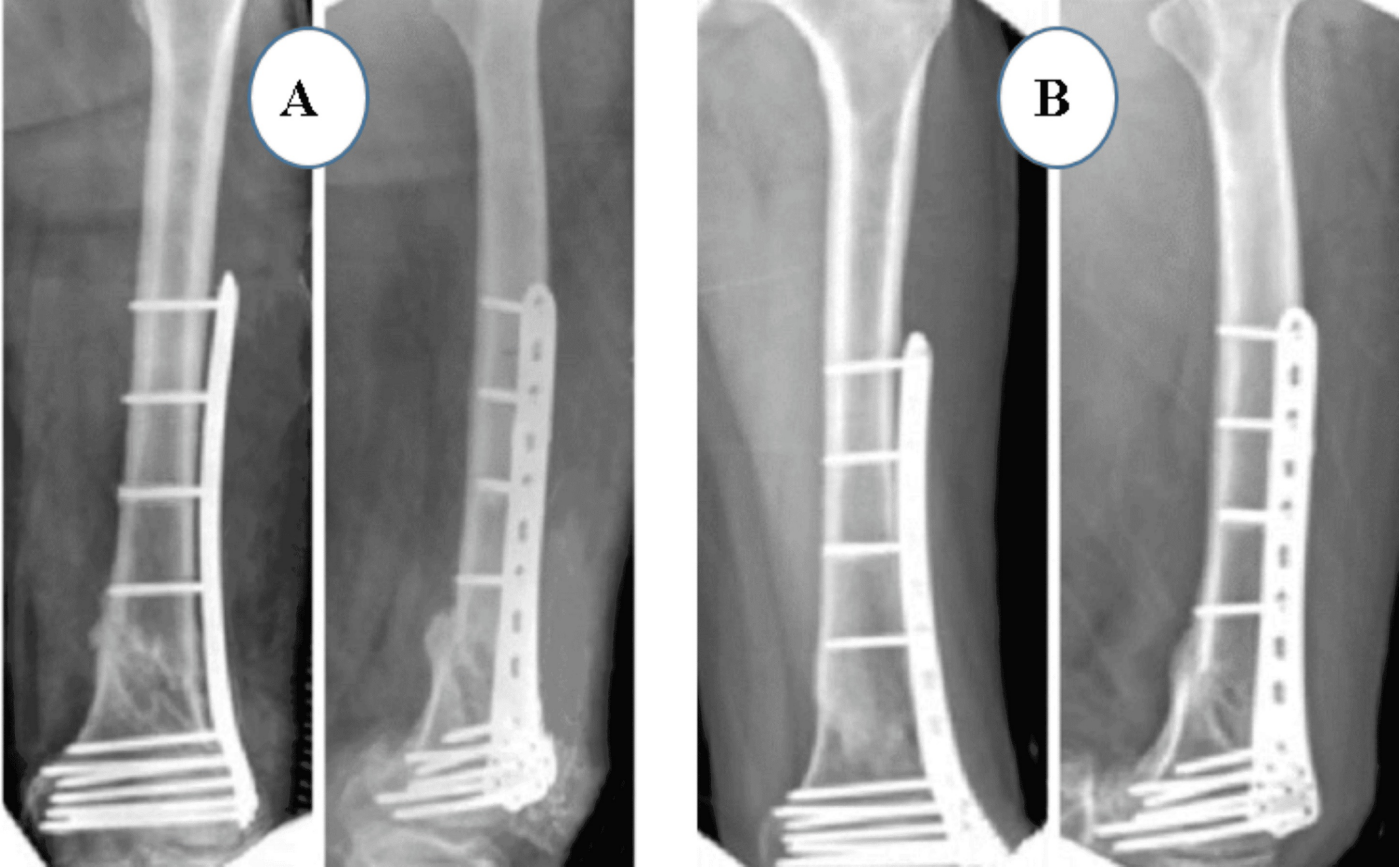
Case 2 follow-up X-rays at (A) three and (B) six months

**Figure 7:**
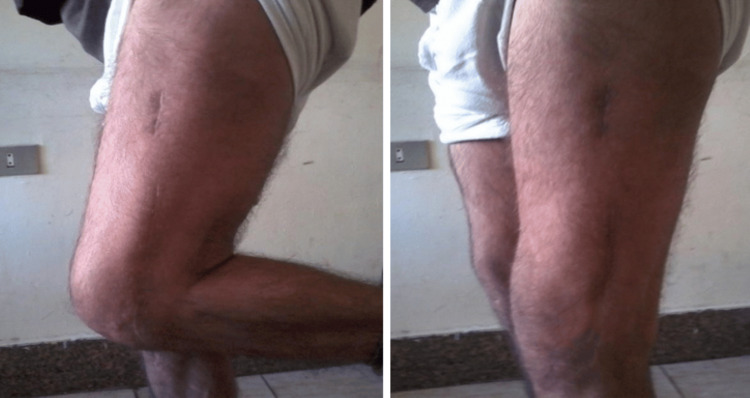
Case 2 ROM from 0° full extension to 90° flexion at six months postoperatively ROM: range of motion

Postoperatively (Figures 5-6), passive and active ROM exercises were initiated on the fourth postoperative day. Knee joint alignment was satisfactory, showing approximately 10° of valgus without rotational deformity. Physiotherapy was commenced in the third postoperative week; however, the patient was reluctant and did not complete the program, resulting in reduced postoperative ROM. Partial weight-bearing was initiated at around seven weeks. No complications were observed during the recovery period. Radiological union was achieved at approximately nine weeks postoperatively. At follow-up, the patient’s ROM was from 0° of full extension to 90° of flexion (Figure 7), walking was unrestricted, and usual activities were resumed by approximately 21 weeks. The total follow-up period was around six months.

Case 3

**Figure 8:**
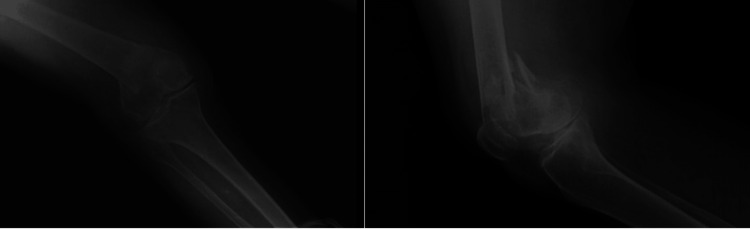
Case 3 preoperative X-rays: anteroposterior and lateral views

The patient was a 61-year-old male who sustained a DFF following a simple fall and slide at home. Preoperative evaluation (Figure 8) revealed a right-sided DFF classified as AO/OTA type A1. The patient had coexisting conditions of diabetes mellitus and hypertension, and no other injuries were associated with the trauma. The interval between injury and surgery was three days.

Operative management involved careful indirect reduction of the fracture under C-arm guidance. Following successful reduction, a DFLP was applied to achieve stable fixation and optimize healing.

**Figure 9:**
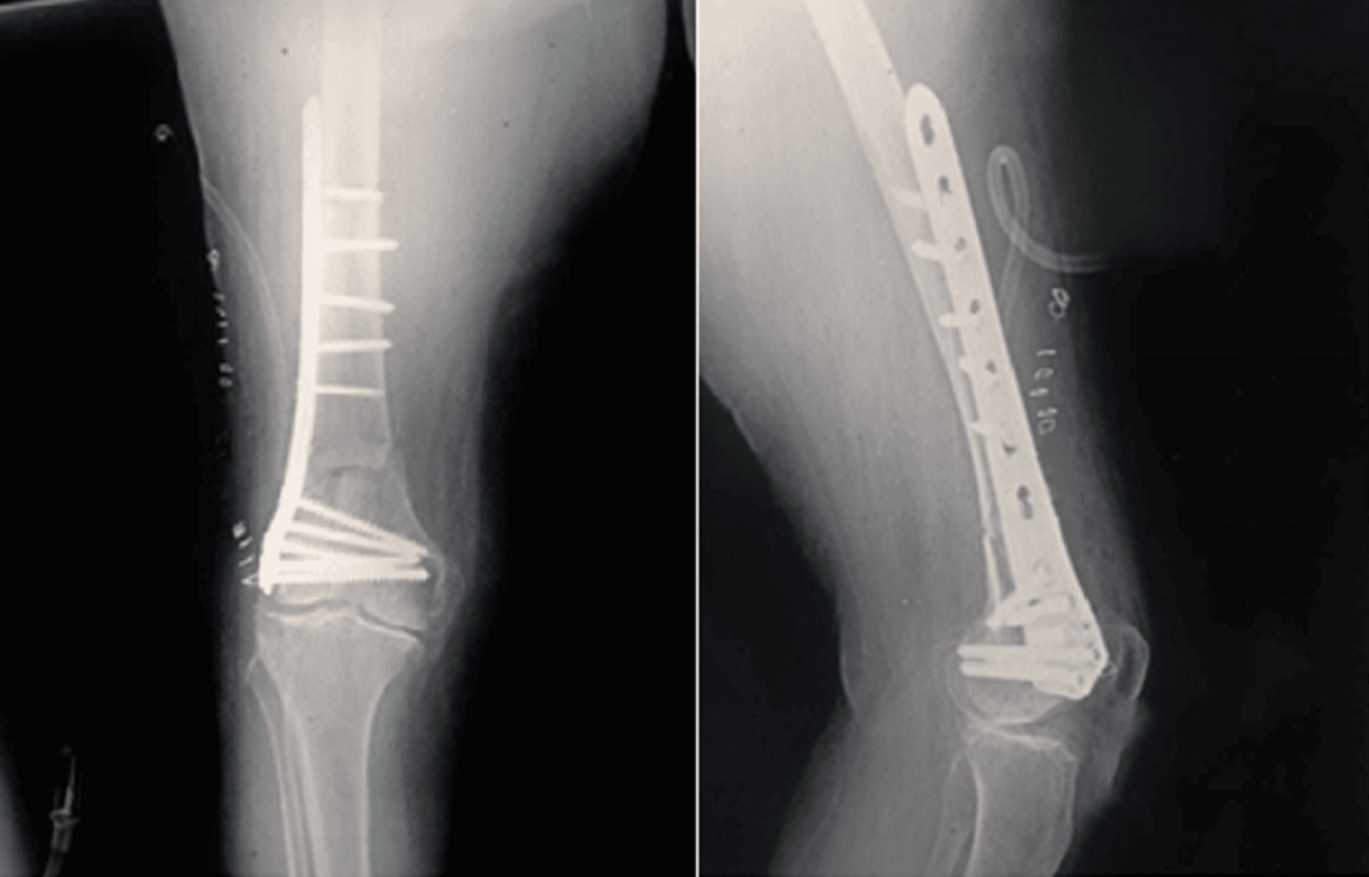
Case 3 immediate postoperative X-rays

**Figure 10:**
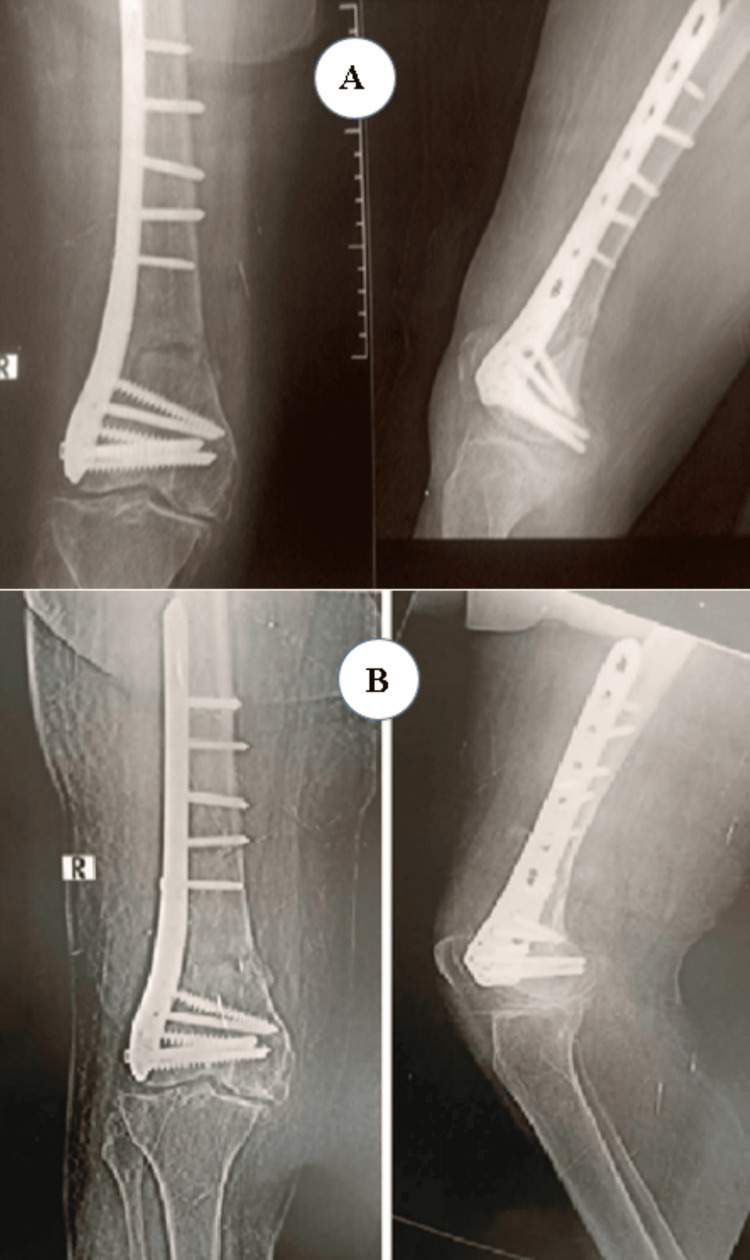
Case 3 follow-up X-rays at (A) three and (B) six months

**Figure 11:**
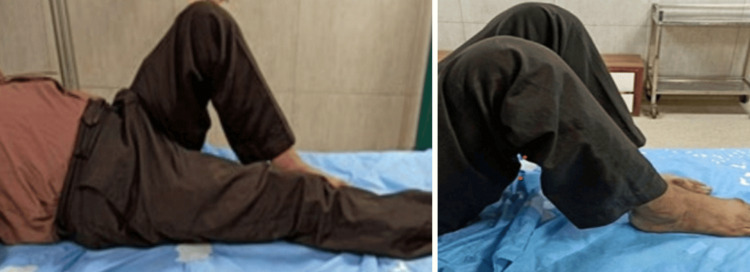
Case 3 ROM from 0° extension to 135° flexion at the 6-month follow-up ROM: range of motion

Postoperatively (Figures 9-11), passive and active ROM exercises were initiated on the second postoperative day. Knee joint alignment was satisfactory, with approximately 7° of valgus and no rotational deformity. Physiotherapy commenced at three weeks postoperatively, and partial weight-bearing was initiated around six weeks. No complications were observed. Radiological union was achieved at approximately nine weeks postoperatively. At follow-up, the patient’s ROM was from 0° of full extension to 135° of flexion, walking was unrestricted, and usual activities were resumed by approximately 20 weeks. The total follow-up period was around six months.

Case 4

**Figure 12:**
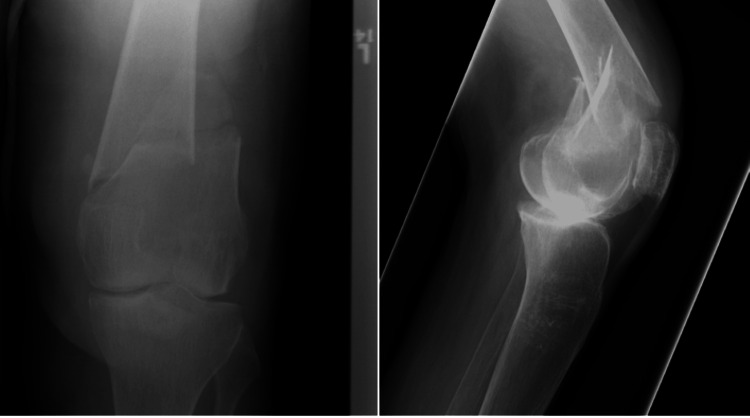
Case 4 preoperative X-rays: anteroposterior and lateral views

The patient was a 40-year-old male who sustained a DFF following a motor vehicle accident. Preoperative evaluation (Figure 12) revealed a left-sided DFF classified as AO/OTA type A1. The patient had no coexisting conditions, and no other injuries were associated with the trauma. The interval between injury and surgery was two days.

Operative management involved careful indirect reduction of the fracture under C-arm guidance, followed by fixation using a retrograde femoral interlocking nail.

**Figure 13:**
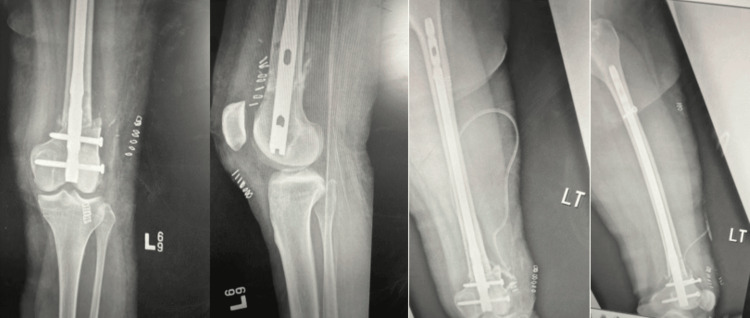
Case 4 immediate postoperative X-rays

**Figure 14:**
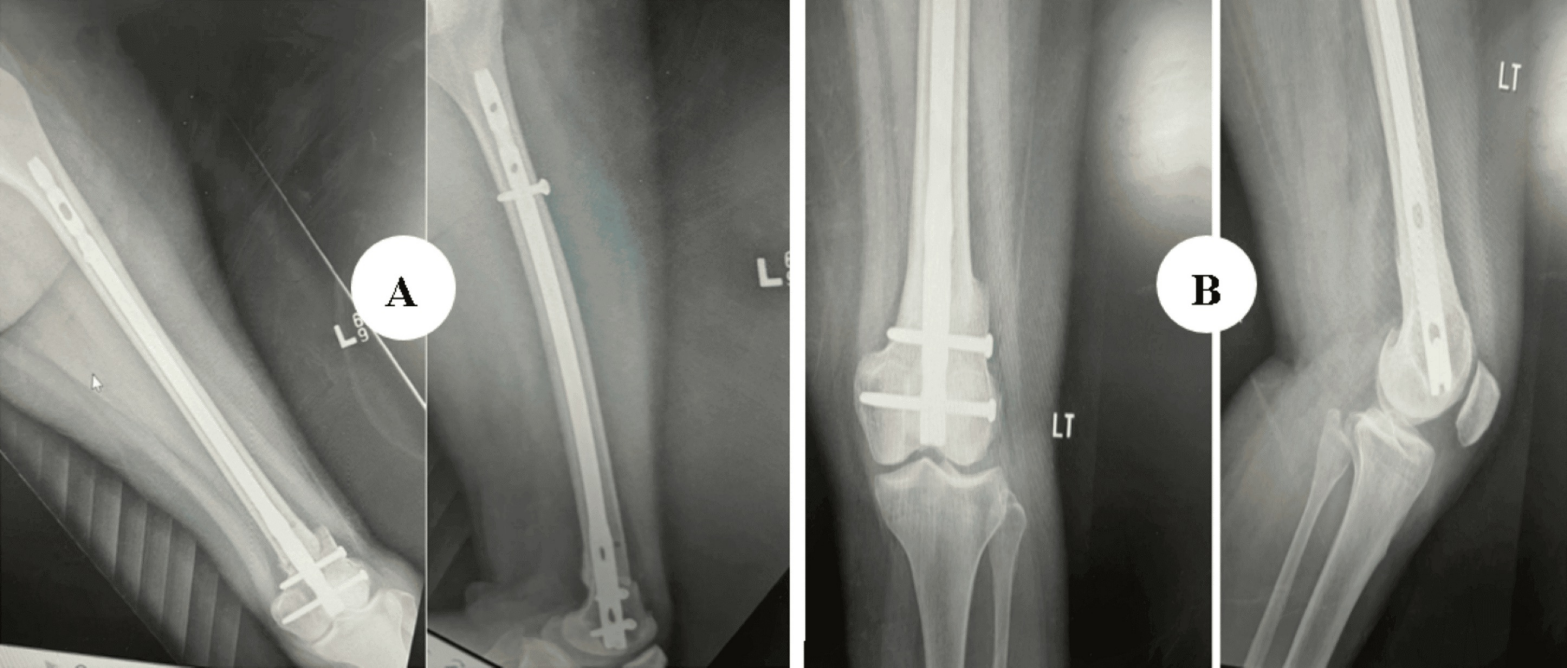
Case 4 follow-up X-rays at (A) three and (B) six months

Postoperatively (Figures 13-14), passive and active ROM exercises were initiated on the second postoperative day. Postoperative radiographs demonstrated a reduced valgus alignment of 2°, as measured by the anatomical lateral distal femoral angle (mLDFA of 92°), which is slightly below the typical physiological valgus range of 5-10°. This alignment remained stable throughout follow-up and did not interfere with fracture healing. Physiotherapy commenced at three weeks postoperatively, and partial weight-bearing was encouraged starting at around four weeks. No complications were observed. Radiological union was achieved at approximately eight weeks postoperatively. At follow-up, the patient’s ROM was from 0° of full extension to 135° of flexion, walking was unrestricted, and usual activities were resumed by approximately 12 weeks. The total follow-up period was around six months.

Case 5

**Figure 15:**
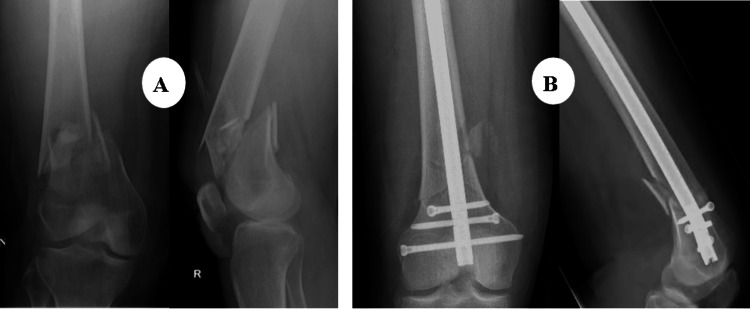
Case 5 (A) preoperative X-rays: anteroposterior and lateral views and (B) immediate postoperative X-rays

The patient was a 36-year-old male who sustained a DFF following a motorbike accident. Preoperative evaluation (Figure 15A) revealed a right-sided DFF classified as AO/OTA type A2. The patient had no coexisting conditions, and no other injuries were associated with the trauma. The interval between injury and surgery was two days.

Operative management involved careful indirect reduction of the fracture under C-arm guidance, followed by fixation with a retrograde femoral interlocking nail.

**Figure 16:**
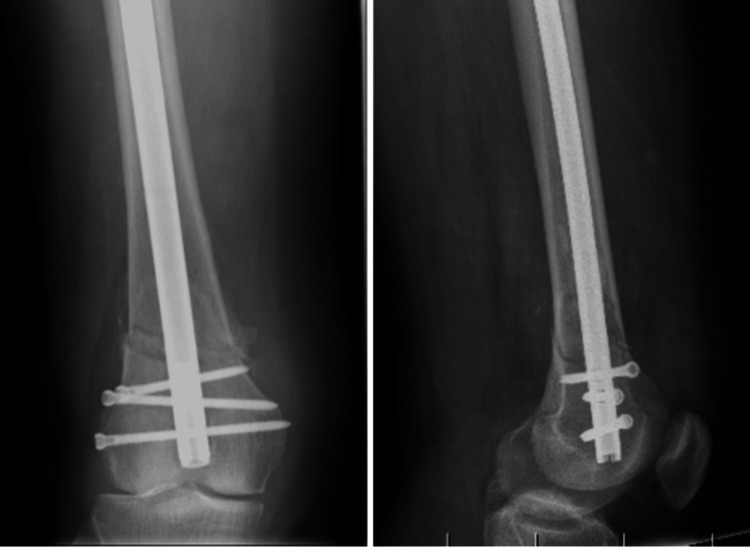
Case 5 follow-up X-ray at six months

**Figure 17:**
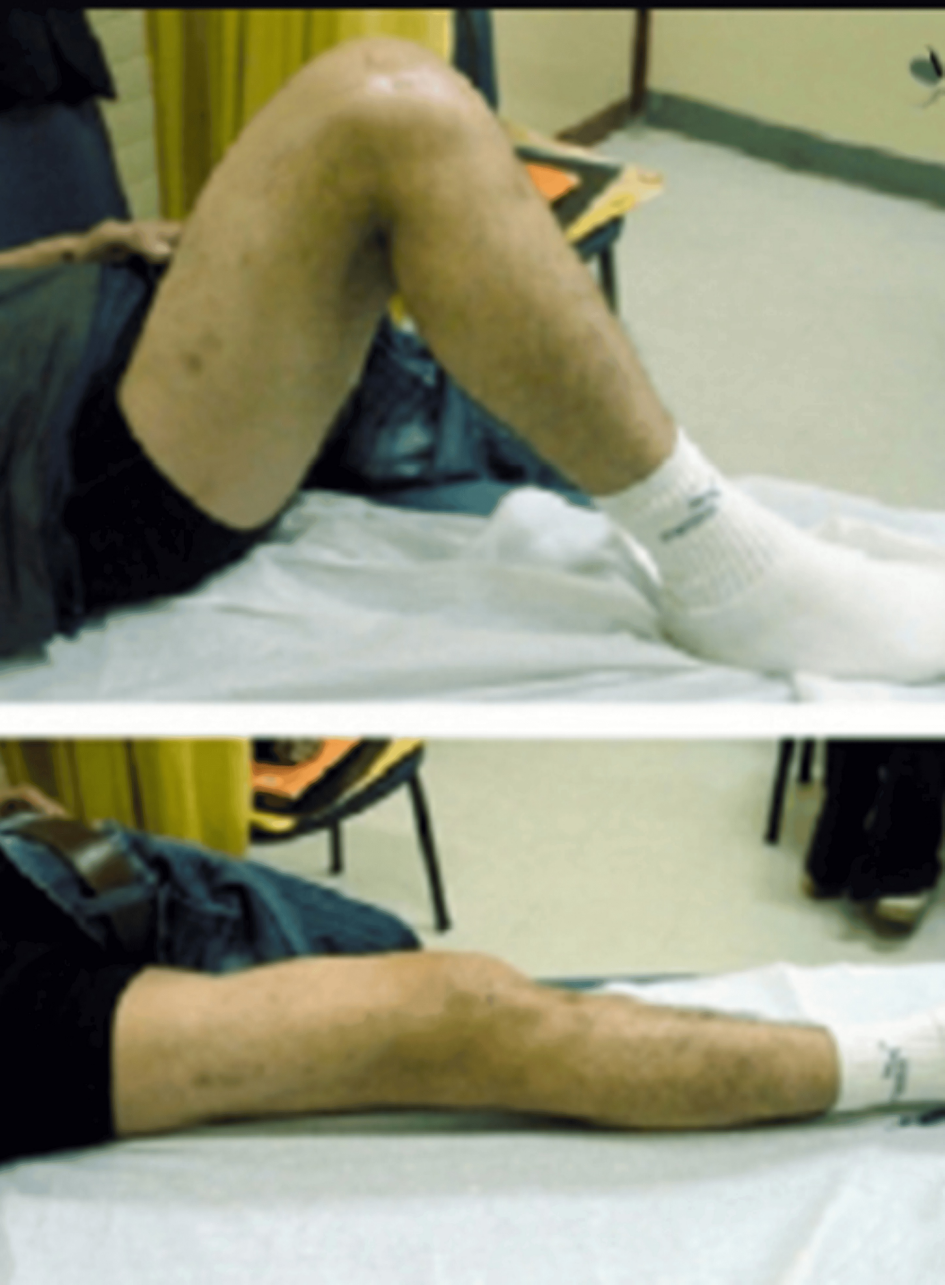
Case 5 ROM from 0° extension to 135° flexion at the six-month follow-up ROM: range of motion

Postoperatively (Figures 15B-17), rehabilitation began on the second day after surgery, focusing on passive and active ROM exercises to promote early joint mobilization. Knee joint alignment was satisfactory, with approximately 7° of valgus and no rotational deformity. Physiotherapy commenced at four weeks postoperatively, while partial weight-bearing was initiated around three weeks. No complications were observed. Radiological union was achieved at approximately eight weeks postoperatively. At follow-up, the patient’s ROM was from 0° of full extension to 135° of flexion, walking was unrestricted, and usual activities were resumed by approximately 12 weeks. The total follow-up period was around six months.

Case 6

**Figure 18:**
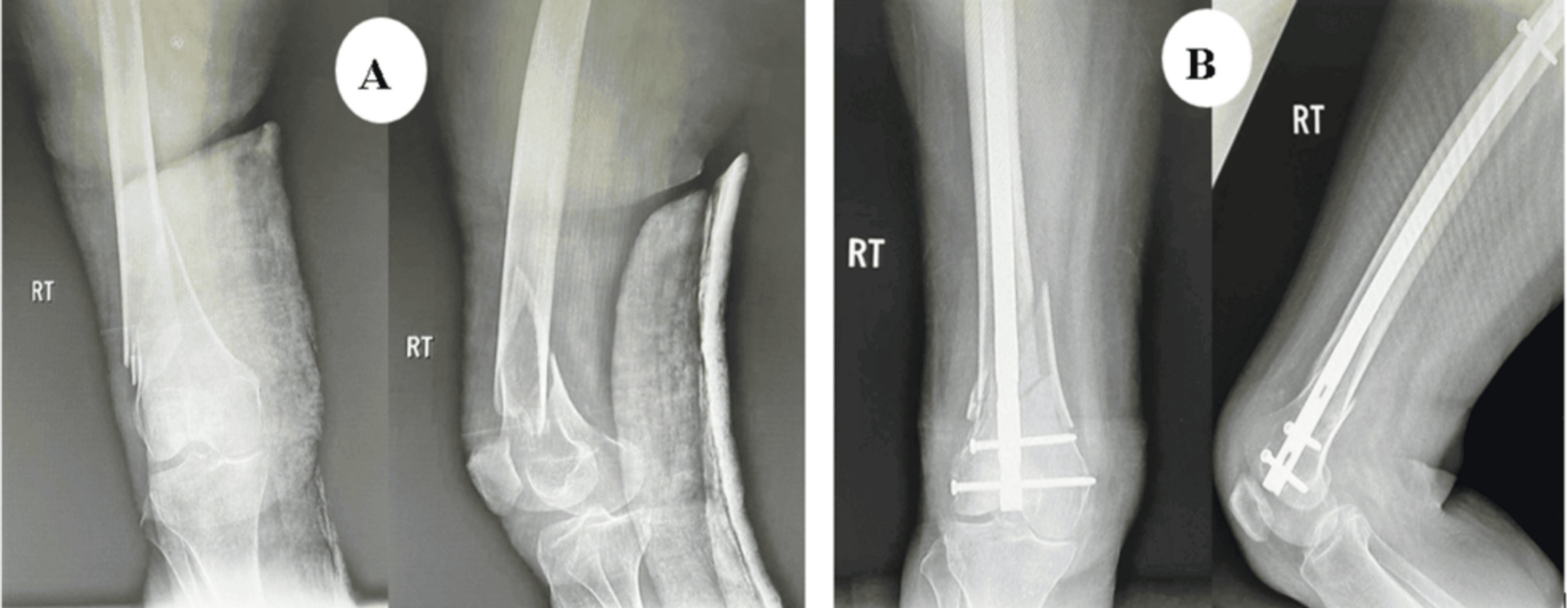
Case 6 A. preoperative anteroposterior and lateral X-rays, B. immediate postoperative X-ray.

The patient was a 72-year-old female who sustained a DFF following a slip and fall. Preoperative evaluation (Figure 18A) revealed a right-sided DFF classified as AO/OTA type A1. She had coexisting conditions of diabetes mellitus and hypertension, and no other injuries were associated with the trauma. The interval between injury and surgery was three days.

Operative management involved careful indirect reduction of the fracture under C-arm guidance, followed by fixation using a retrograde femoral interlocking nail.

**Figure 19:**
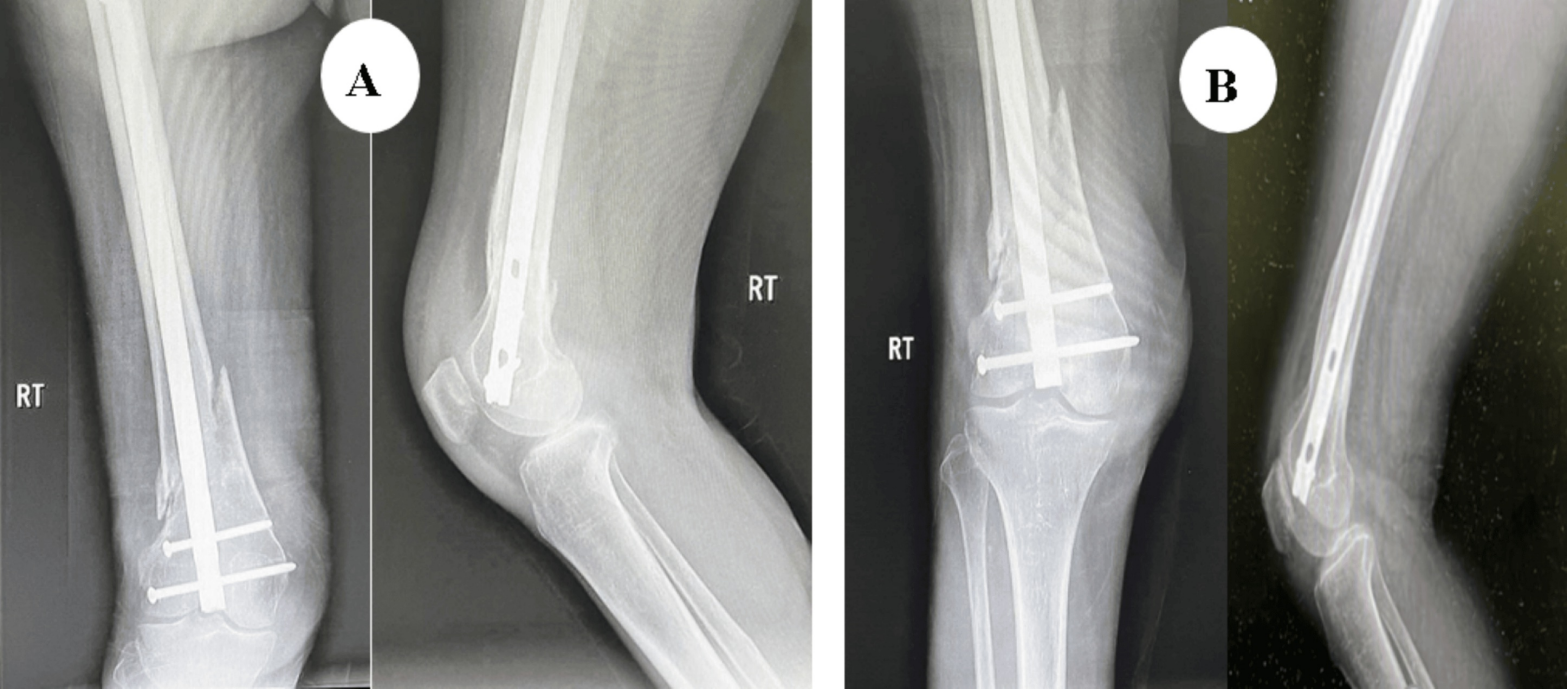
Case 6 follow-up X-rays at (A) three and (B) six months

**Figure 20:**
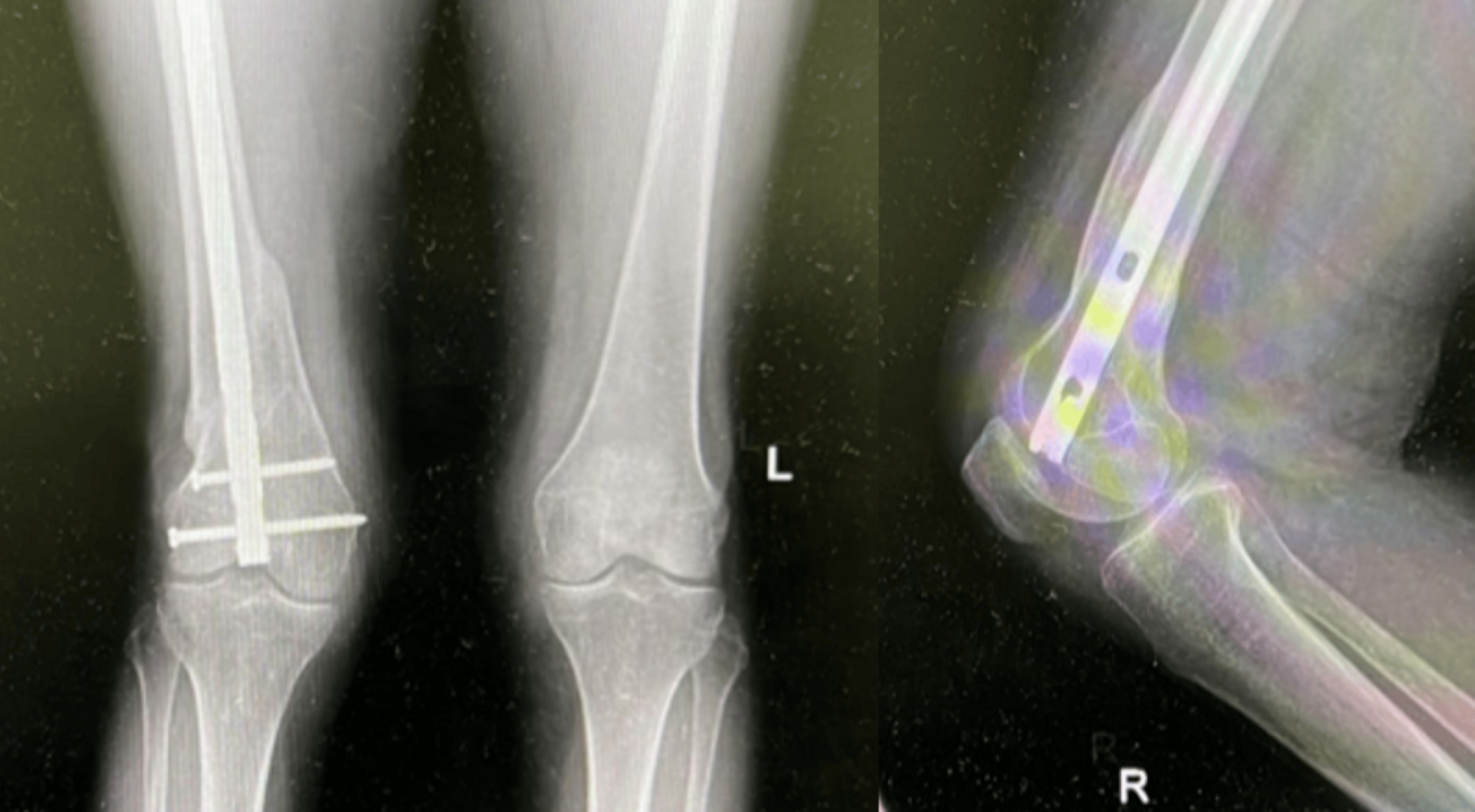
Case 6 X-ray showing complete union of the DFF at eight months DFF: distal femoral fracture

**Figure 21:**
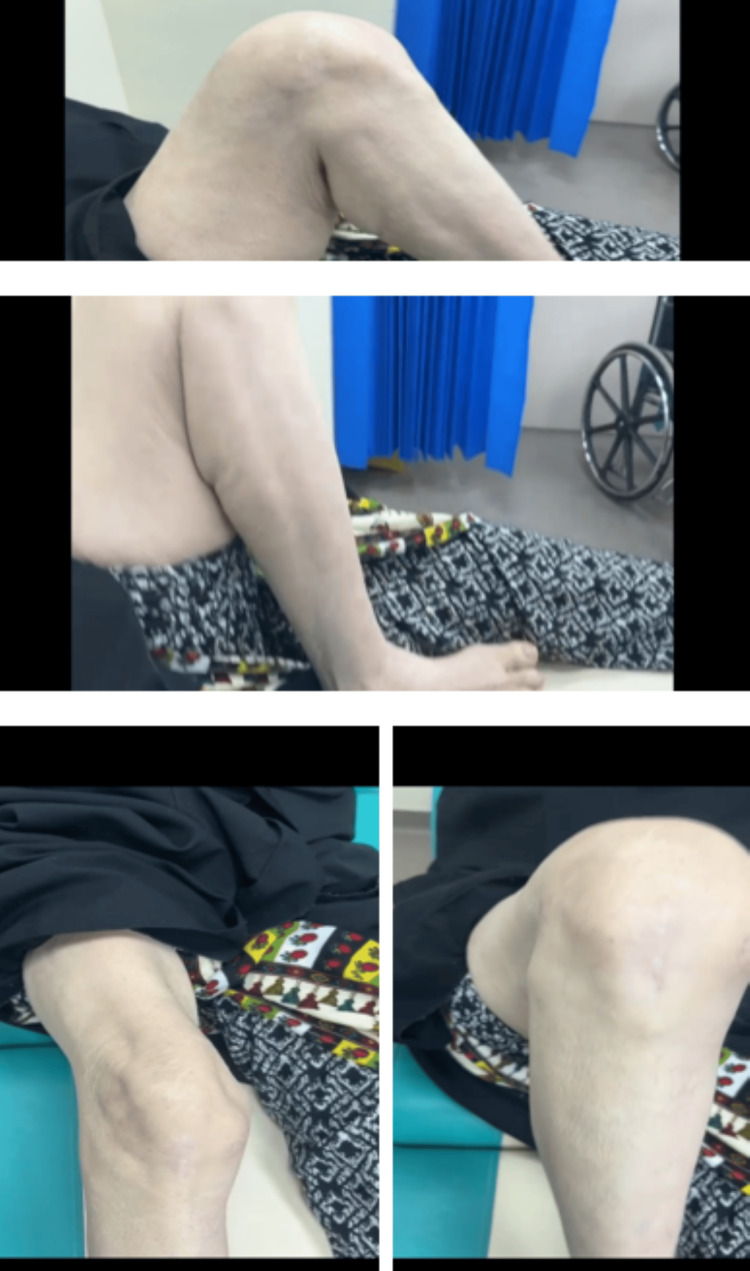
Case 6 knee ROM from 0° extension to 135° full flexion ROM: range of motion

Postoperatively (Figures 18B-20), passive and active ROM exercises were initiated on the fifth postoperative day. Knee joint alignment was satisfactory, with approximately 12° of valgus and no rotational deformity. Physiotherapy commenced at four weeks postoperatively, and partial weight-bearing was encouraged around six weeks. Radiological union was initially noted at approximately 12 weeks; however, the patient experienced delayed union, with full union achieved at eight months. At follow-up, her ROM was from 0° of full extension to 135° of flexion (Figure 21). Walking was initially restricted with a walker frame, and she returned to normal activities by approximately 21 weeks. The total follow-up duration exceeded six months.

## Discussion

DFFs remain a challenge to manage because of their complex anatomy, comminution, and frequent association with osteoporosis and, in elderly patients, pre-existing knee arthritis. Historically, conservative treatment such as traction and casting often resulted in complications, including pneumonia, thromboembolism, joint stiffness, and poor functional outcomes [7]. Operative fixation has therefore become the preferred approach. However, the risk of infection, delayed union, and non-union persists [8]. This prospective comparative study was designed to address a gap in the literature by focusing exclusively on a homogenous cohort of extra-articular (AO/OTA 33-A) DFFs, a population often combined with intra-articular patterns in previous studies. Our findings demonstrate that both MIPO and RIMN are effective fixation strategies for these fractures, with each method presenting a distinct profile of advantages.

MIPO presents numerous theoretical advantages over the traditional open plating approach. Fracture‑bridging osteosynthesis can confer robust mechanical stability without resorting to extensive periosteal stripping or imposing undue surgical trauma upon the osseous substrate and surrounding soft‑tissue envelope. By preserving the fracture’s intrinsic vascular network and maintaining the osteogenic hematoma, the biological milieu conducive to bone repair remains intact [9].

Anatomical locked plates (LPs) are intended for placement on the lateral distal femoral cortex; secure fixation in a locking configuration is achieved by placing a minimum of three bicortical screws in the distal fragment and at least four cortices of purchase proximal to the fracture. The main objective of intervention with LPs is to achieve union by bridging the callus through relative stability, thereby permitting mobility within the fracture site. That biomechanical concept of relative stability facilitates dynamic deformation, resulting in secondary callus formation, whereas absolute stability promotes primary callus formation [10].

Retrograde IMNs constitute a highly efficacious operative strategy for DFFs, obviating extensive soft‑tissue dissection and thereby preserving the peri‑fracture vascular environment. Biomechanical analyses have demonstrated that these nails provide substantially greater construct stiffness and markedly attenuate interfragmentary micromotion under axial loading relative to DCS or locked condylar plates, facilitating earlier functional rehabilitation. In osteoporotic bone, especially when comminution is pronounced, secure fixation poses a formidable challenge; here, the design and application of distal locking screws critically augment implant stability [11].

Based on the NEER criteria, an investigation found that 80% of cases in group I (MIPO plating) achieved excellent outcomes, while 20% had satisfactory results, with no poor outcomes. In group II (RIMN nail), 30% of cases achieved excellent results, 66% satisfactory outcomes, and 4% unsatisfactory outcomes. This investigation was comparable to the prior one conducted by Helfet and Lorich [12]. Closed RIMN and a less invasive plating system were both effective and safe options for treating extra-articular DFFs. Still, the MIPO technique had fewer side effects [6].

The management of extra-articular DFFs continues to generate debate regarding the optimal fixation method. In this prospective comparative cohort study, we found no statistically significant differences between MIPO and RIMN regarding union rates and overall functional outcomes; however, each technique demonstrated distinct advantages and drawbacks.

This study contributes prospective data comparing MIPO and RIMN exclusively in extra-articular (AO/OTA 33-A) DFFs, focusing on functional outcomes and rehabilitation timelines. Unlike many earlier series that focused primarily on radiographic union, this study prospectively measured validated functional outcomes (KSS and LKS) and reported complication risks with RRs and 95% CIs, providing clinicians with more interpretable effect sizes.

Both groups achieved high union rates and satisfactory knee function within six months. These findings align with prior studies by Mahar et al. [6], Markmiller et al. [13], and Henderson et al. [14], which reported comparable outcomes between plates and nails. Results were graded according to KSS. LKS did not show a significant advantage of one technique over the other, as 50% of cases showed excellent results, 30% showed promising results, 10% were fair, and 10% were poor in group I (MIPO plating). In group II (interlocking nail), 50% of cases showed excellent results, 35% good, 10% fair, and 5% poor. The infection rate in this series was low; there were only two cases of superficial infection in the first group and none in the second.

Age, sex, side affected, mechanism of injury, occupation, and concomitant diseases did not have a significant effect on the results; in addition, hospital stay and the rate of unsatisfactory results did not show a marginal deviation between methods.

Radiological union is identified by bridging callus development across three cortices. The present investigation observed an average time to union of approximately 14 weeks, consistent with the observations of Markmiller et al. and Henderson et al., who reported 14 and 12 weeks, respectively [13,14]. A similar interval of 11-14 weeks was noted by other authors as well [15,16]. The average union time for nailing was 15 weeks, which aligns with the observations of Kumar et al. [16], Gellman et al. [17], and Ingman [18], who reported durations of 12, 14, and 12 weeks, respectively.

Most importantly, our findings demonstrate no statistically significant difference in the time to radiological union between the two techniques (14.3 vs. 15.1 weeks, p = 0.214). Furthermore, no patient in either group required a reoperation. These results provide strong evidence that both MIPO and RIMN are equally reliable and effective fixation strategies for achieving the primary goal of fracture healing in extra-articular DFFs (AO/OTA 33-A), with a similarly low risk of failure necessitating revision surgery. It is important to distinguish radiographic ideals from clinical success; the few cases with alignment outside the optimal range (all in the RIMN group) successfully achieved the primary treatment goals of union, stability, and functional recovery.

The significantly earlier full weight-bearing and greater knee flexion observed in the RIMN group can be attributed to the intramedullary implant's inherent axial stability, which permitted a more aggressive rehabilitation protocol. This protocol difference, which reflects the distinct biomechanical properties of each fixation method, is crucial for interpreting the functional outcome disparities between the groups.

RIMN was associated with a higher mean knee flexion at final follow-up (112° vs. 107°). The mean difference of 5.0° was statistically significant (95% CI 1.2 to 8.8; t(38) = 2.68, p = 0.011), indicating greater flexion after nailing in our cohort. This difference may be attributed to the earlier initiation of knee mobilization in the nailing cohort. Kumar et al. [16], Gellman et al. [17], Ingman [18], and Lucas et al. [19] documented average knee flexion of 100°, 106°, 101°, and 104°, respectively, following the implementation of nailing techniques. However, Markmiller et al. [13], Schutz et al. [15], and Kregor et al. [20] noted average knee flexion of 110°, 107°, and 103° after plating. We hypothesize that the earlier weight-bearing and ambulation permitted by RIMN facilitate more rapid strengthening of the knee musculature, which may contribute to this observed difference in ROM. However, as noted in the study limitations, the sample size precludes a definitive conclusion on this relationship.

Group I experienced fewer complications than group II; however, the results were comparable between the two groups. Our results were comparable to some previous investigations [21,22]. The observed superficial infection rate (10%) in the MIPO group was consistent with published ranges and was generally attributable to lateral incisions and soft-tissue handling. Conversely, anterior knee pain was more prevalent (20% vs. 5%) after nailing than after plating, with similar findings in other series: Shroff and Bhamare reported a higher incidence of knee pain following nailing (22.22%) compared to plating (8.88%). The higher incidence of anterior knee pain after RIMN is consistent with previous reports [23]. It is likely related to the intra-articular starting point and implant prominence, rather than greater postoperative mobility. Taken together, these results emphasize a practical trade-off: RIMN permits earlier mobilization and slightly greater early knee flexion (beneficial for younger, high-demand patients), while MIPO is associated with lower rates of knee morbidity and may be preferable in older or osteoporotic patients seeking to minimize anterior knee problems.

Overall, our results confirm that both MIPO and RIMN are safe and effective for extra-articular DFFs. MIPO may reduce knee-related morbidity, whereas RIMN offers faster rehabilitation. These findings support the view of earlier comparative studies [6,13,21,22], while highlighting the need for longer follow-up to assess implant survival and late complications.

Although both fixation methods are well-established, our findings refine patient selection criteria by quantifying trade-offs in knee pain and mobility, which are rarely reported in earlier comparative studies.

Limitations

This study has several limitations. First, the sample size was modest (n = 40), which restricts subgroup analysis and reduces power to detect small differences. Consequently, clinically relevant trends, such as the fourfold higher incidence of anterior knee pain in the RIMN group, may not have reached statistical significance due to this limited power. However, this number is comparable to those reported in many published prospective series on DFF fixation (e.g., Mahar et al., n = 50; Markmiller et al., n = 23). Second, the follow-up period of six months is relatively short for evaluating late complications such as implant failure or post-traumatic arthritis. Third, the non-randomized allocation (surgeon clinical judgment) introduces potential selection bias; we attempted to mitigate this by enrolling consecutive patients and comparing baseline characteristics between groups, but residual confounding may remain. These limitations temper causal inferences from our findings and indicate that larger, multicenter studies with longer follow-up would be valuable.

Furthermore, our postoperative protocol employed a cautious approach to weight-bearing, which may not align with the modern trend toward immediate weight-bearing in stable fixations. This conservative protocol likely influenced the measured time to full weight-bearing and should be considered when interpreting the rehabilitation outcomes. Finally, as noted in the discussion, the modest sample size precludes definitive conclusions regarding the observed trend of superior knee ROM after RIMN, an area that warrants future investigation. These limitations temper causal inferences from our findings and highlight the need for larger, multicenter randomized trials with longer follow-up.

## Conclusions

Both MIPO and RIMN yielded comparable, favorable radiological and functional outcomes for extra-articular distal femoral fractures in this prospective cohort. RIMN was associated with earlier progression to full weight-bearing and a trend toward greater knee ROM. In contrast, MIPO was associated with lower rates of anterior knee pain in this series. Clinical decision-making should be individualized based on fracture pattern, bone quality, and patient priorities: RIMN may be favored for earlier rehabilitation in mobile patients, whereas MIPO may be preferred when minimizing knee morbidity is essential. Larger randomized studies with longer follow-up are needed to confirm these findings.
